# Supplementary material for: Enhanced Surface Plasmon by Clusters in TiO2-Ag Composite
Source: Materials (Basel). 2022 Oct 26;15(21):7519. doi: 10.3390/ma15217519 (PMC9657337; doi:10.3390/ma15217519)
Supplement: Supplementary file 1 [file materials-15-07519-s001.zip › materials-1940994-supplementary.pdf]

materials

Article

Enhanced Surface Plasmon by Clusters in TiO_2-Ag Composite

Yongjun Zhang [1], Zhen Xu [1], Shengjun Wu [2,*], Aonan Zhu [3], Xiaoyu Zhao [1] and Yaxin Wang [1,*]

[1] School of Material and Environmental Engineering, Hangzhou Dianzi University, Hangzhou 310018, People's Republic of China
[2] Department of Clinical Laboratories, Sir Run Run Shaw Hospital, School of Medicine, Zhejiang University, Hangzhou 310020, People's Republic of China
[3] College of Chemistry, Nankai University, Tianjin 300071, People's Republic of China
* Correspondence: 3202107@zju.edu.cn (S.W.); yaxinwang@hdu.edu.cn (Y.W.)

Supplementary information

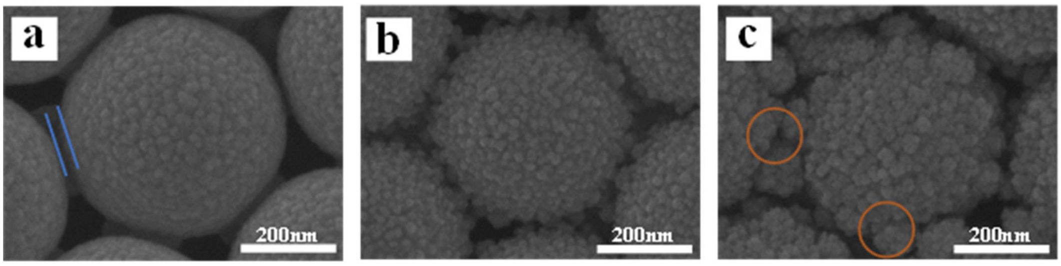

Figure S1. (**a**–**c**) SEM images for the samples with TiO_2 sputtering power 20 W, 40 W and 60 W respectively and Ag sputtering power of remains unchanged.

SEM images confirms the low concentration of TiO_2 addition has little effect on Ag nanocaps into some and Ag nanoparticles are still compact under SEM observations. More TiO_2 addition leads to the coarse surface and finally results in the cluster decorated TiO_2 -Ag nanocaps.

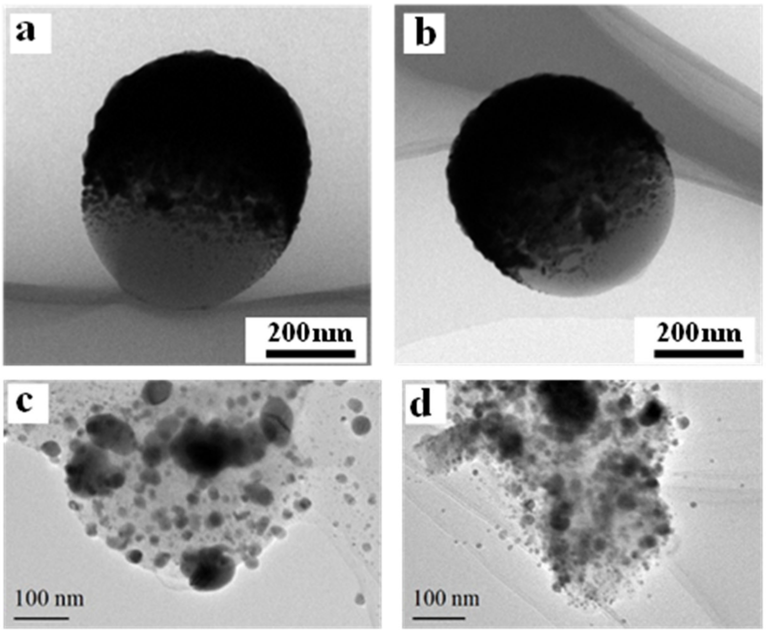

Figure S2. (**a**) TEM image for Ag nanocaps; (**b**–**d**) TEM images for the samples with TiO_2 sputtering power 20W, 40W and 60W respectively.

Citation: Zhang, Y.; Xu, Z.; Wu, S.; Zhu, A.; Zhao, X.; Wang, Y. Enhanced Surface Plasmon by Clusters in TiO_2-Ag Composite. *Materials* **2022**, *15*, 7519. https://doi.org/10.3390/ma15217519

Academic Editor: Sergio Morales-Torres

Received: 13 September 2022
Accepted: 24 October 2022
Published: 26 October 2022

Publisher's Note: MDPI stays neutral with regard to jurisdictional claims in published maps and institutional affiliations.

Copyright: © 2022 by the authors. Licensee MDPI, Basel, Switzerland. This article is an open access article distributed under the terms and conditions of the Creative Commons Attribution (CC BY) license (https://creativecommons.org/licenses/by/4.0/).

TEM observations show the nanoparticles of Ag nanocaps are still compact under as shown in Figure S2a. The low concentration of TiO_2 addition, for example, sputtering power 20W, shows little effects on Ag nanocaps, which is quite similar to Ag nanocaps, as shown in Figure S2b. More TiO_2 addition results in smaller Ag nanoparticles, for example, the sputtering power 40W results in Ag nanoparticles with the size around 25nm as shown in Figure S2c, and the sputtering power 60W results in Ag nanoparticles with the size around 15nm, as shown in Figure S2d.
